# The Influence of Tool Geometry on the Mechanical Properties and the Microstructure of AA6061-T6 Aluminum Alloy Friction Stir Spot Welding

**DOI:** 10.3390/ma16114135

**Published:** 2023-06-01

**Authors:** Amir Alkhafaji, Daniel Camas, Pablo Lopez-Crespo, Hayder Al-Asadi

**Affiliations:** 1 Department of Civil and Materials Engineering, University of Malaga, E-29071 Malaga, Spain; dcp@uma.es (D.C.); plopezcrespo@uma.es (P.L.-C.); 2 Sewerage Directorate, Nasiriyah 6400, Iraq; h.k.alasadi@gmail.com

**Keywords:** FSSW, shoulder diameter, pin profile

## Abstract

In this work, the influence of the tool geometry on friction stir spot welding (FSSW) was studied on sheets made of AA6061-T6 aluminum alloy. Four different AISI H13 tools with simple cylindrical and conical pin profiles and 12 mm and 16 mm shoulder diameters were used to perform the FSSW joints. Sheets of 1.8 mm thickness were used during the experimental work to prepare the lap-shear specimens. The FSSW joints were performed at room temperature. For each joining condition, four specimens were carried out. Three specimens were used to find the value of the average tensile shear failure load (TSFL), while the fourth one was used to examine the micro-Vickers hardness profile and to observe the microstructure of the cross-section of the FSSW joints. The investigation concluded that higher mechanical properties corresponding to the finer microstructure were obtained by the conical pin profile and the higher shoulder diameter compared with the specimens performed using the cylindrical pin tool and lower shoulder diameter due to the higher strain hardening and the higher frictional heat generation, respectively.

## 1. Introduction

**Figure 1:**
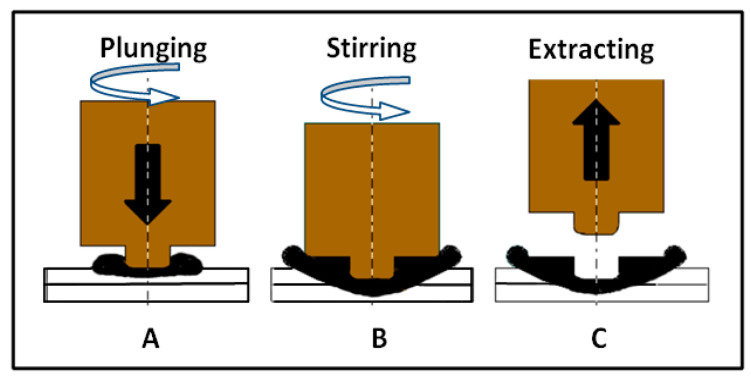
A schematic diagram of FSSW process.

Recently, aluminum alloys have become very attractive in research, industry, and design as promising materials for structures of automotive industry and aerospace applications. Specifically, 6xxx alloys have been studied extensively for their better strength, formability, corrosion resistance, and relatively lower cost [1]. Friction stir welding (FSW) is a technique for joining similar and dissimilar light weight metals and alloys such as aluminum, copper, zinc, titanium, and magnesium alloys in solid state. FSW was first invented in 1991 by The Welding Institute (TWI) in the UK and has been employed in aerospace, rail, automotive, and marine industries [2,3]. FSW avoids some fusion welding defects, such as porosity, fume, and cracking with low residual stresses, improved dimensional stability, good mechanical properties, and high surface finish [4]. FSW can join all of the aluminum alloys from the 2XXX to the 7XXX series that had not been considered weldable alloys using conventional welding methods because of the excessive decrease in joint strength compared to the base material. FSW can produce many configurations, such as butt, lap, and T-joints, which are widely used in assemblies of parts in aircraft and automotive industries [5]. Friction stir spot welding (FSSW), as a derivative of FSW, was developed by Mazda Motor Corporation and Kawasaki Heavy Industry [6]. It is a method for joining lightweight metals in solid state. The FSSW process consists of three distinct steps as shown in Figure 1. Step A, a rotating non-consumable tool with a shoulder and pin is plunged through the upper sheet into the lower sheet to a desired depth until the tool shoulder contacts the top of the upper sheet. During the second step, B, the tool is then held into the workpiece sheets briefly before its retraction in step C. The tool pin makes a characteristic hole called the “keyhole” in the middle of the FSSW weld joint [7]. FSSW has made the spot joining of unweldable, hard-to-weld light alloys, or advanced high-strength steels easy. These materials are the most common ones in the automotive and aerospace industries. Additionally, there are savings of about 90% and 40% in power and equipment use, respectively, compared to resistance spot welding (RSW) [8]. The heat-treatable aluminum 6xxx alloys have moderately high strength levels, better corrosion resistance than the 2xxx and 7xxx alloys, good weldability, and superior extrudability. With yield strength comparable to mild steel, AA6061 is one of the most widely used aluminum alloys [9,10]. AA6061 alloy is one of the most appropriate choices for the light-skin of planes. Among aluminum alloys, aluminum-magnesium-silicon (Al-Mg-Si) heat-treatable alloys, despite medium strength, seem to have a high weldability advantage over high-strength aluminum alloys. That is why Al-Mg-Si alloys are commonly used for structural parts in welded assemblies [11].

The FSSW joint strength is affected by welding parameters, such as tool rotational speed, dwell time, plunge depth, and plunge rate [12]. The tool rotation generates frictional heat due to the contact with the workpiece sheets during the dwell time of the FSSW process. Therefore, tool rotational speed and dwell time play a significant role in the heat generation required for material softening during the FSSW process. Tool geometry affects the contact area and affects the heat generation in the weld nugget. 

Tool geometry mainly includes a pin and a shoulder [13]. It controls the frictional heat generation, the joint formation, and the FSSW joint strength. The pin profile is crucial in material flow, so it controls the welding formation. The tool shoulder generates most of the frictional heat, provides the forging pressure, and prevents the softened material from escaping, while both the pin and the shoulder influence the material flow [14].

The microstructure of the weld zones includes the stir zone (SZ) adjacent to the keyhole. This area contains fine equiaxed grains, resulting from higher heat and plastic deformation; therefore, dynamic recrystallization occurs in this zone. The microstructure of the weld zone also includes the thermomechanically affected zone (TMAZ), which contains lower fine grains in the surrounding areas of the SZ and the heat-affected zone (HAZ), which is only affected by the heat that lies between the TMAZ and the base metal (BM), and has a coarser grain size than the BM due to the effect of frictional heat generated during the FSSW process. 

Several studies have investigated the effect of tool geometry on the mechanical properties of the FSSW of aluminum alloys. For example, Bilici et al. [13] reported that the tapered pin produced higher welding force than the threaded straight cylindrical pin during their study of the pin profile and shoulder geometry effects in friction-stir-spot-welded polymer sheets [13]. Abbass et al. [15] reported that cylindrical pin profiles produced the highest shear load in FSSW joints when compared with threaded cylindrical with flute and tapered cylindrical pin tools in their study of dissimilar FSSW for aluminum alloy AA2024T3 and commercially pure copper sheets. Bilici, [16] observed that the greatest tensile strength was obtained with a threaded cylindrical pin tool compared to straight cylindrical and square pin tools in his investigation on FSSW of polypropylene sheets. D. H. Choi et al. [17] reported that, under the various welding parameters applied in their study, the cylindrical-shaped tool with small projection (CTP) demonstrated superior mechanical properties to those of the threaded pin tool (TPT) and the simple cylindrical tool (CT). Therefore, suitable tool design can improve weld quality and weld joint strength. Additionally, tool material should be strong and hard and present enough corrosion resistance to avoid tool wear during FSSW and to produce a successive FSSW process. Some researchers have shown that hot-working tool steels, for example, those (AISI H13) with thicknesses between 0.5 and 50 mm, are suitable for welding aluminum alloys [18]. 

A few studies have investigated the influence of tool geometry in friction stir spot welding on the mechanical properties of aluminum alloys, especially the 6XXX series. Therefore, more new research is required to optimize tool geometry in FSSW. 

In this study, the FSSW process was performed in joining AA6061-T6 aluminum alloy sheets to investigate the effect of tool geometry on the mechanical properties and microstructure of the FSSW joints of this alloy with two goals. The first goal was to investigate the effect of the tool shoulder diameter, which controls the frictional heat generation in the weld nugget, and the second goal was to investigate the effect of the tool pin profile, which influences the plastic deformation of the material of the workpiece during the FSSW process.

## 2. Experimental Details

### 2.1. Materials

In this study, sheets of AA6061-T6 aluminum alloy with a thickness of 1.8 mm were used as a base metal of the lap-shear specimens for FSSW joints. Their chemical composition is shown in Table 1, and the mechanical properties of AA6061-T6 aluminum alloy are listed in Table 2.

**Figure 2:**
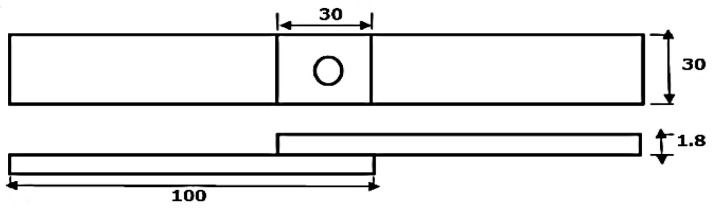
Schematic illustration of the prepared FSSW lap-shear specimens. (Dimensions are mm).

A classic milling machine (Knuth, UWF 12B, Wasbek, Germany) was prepared and employed to carry out the experiment of FSSW for AA6061-T6 aluminum alloy sheets with dimensions of 100 mm length and 30 mm width, prepared in lap configuration to produce lap-shear specimens shown in Figure 2, with an overlap area of 30 × 30 mm^2^. During sample preparation, the sheet cutting direction was kept the same as in the rolling stage of alloy formation in order to load them in the same direction during testing [19]. All the FSSW welds were performed in lap configuration in the centre of the overlap area.

**Figure 3:**
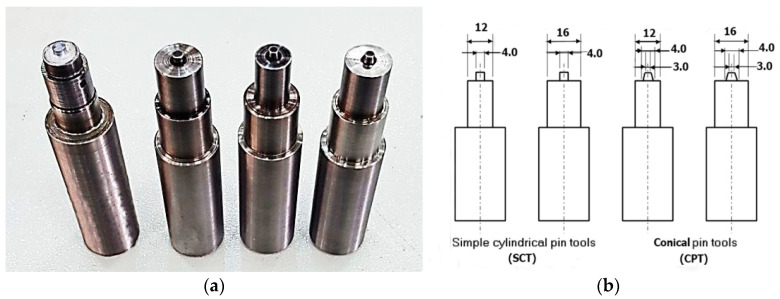
The FSSW tool used: (**a**) simple cylindrical pin tools and conical pin tools; (**b**) schematic diagram of the FSSW tools with constant pin length of 2.6 mm.

Two types of tool pin profiles were used in this investigation, the simple cylindrical pin tool (SCT) and the conical pin tool (CPT), with two values of shoulder diameter—12 mm and 16 mm for each tool pin profile with a constant pin length of 2.6 mm and a pin diameter of 4 mm. Figure 3a shows the tools used in this study, while Figure 3b illustrates a schematic diagram of the used tools with all dimensions. The tool material used in this study was AISI H13 tool steel and it was hardened to 48–50 HRC. The prepared alloy sheets of the lap-shear specimens were cleaned with acetone for the removal of oil and dirt. Fixtures and spacers were used to ensure fixed and exact lap-shear specimens for the FSSW process.

### 2.2. Test Procedures

**Figure 4:**
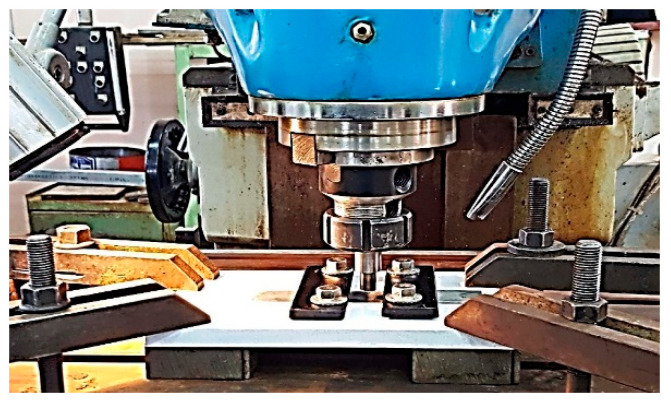
The FSSW process.

All the FSSW welds were performed at the predetermined process parameters, a constant tool rotation speed of 426 rpm, dwell time of 15 s, plunge depth of 0.4 mm, and plunge rate of 15 mm/min. The effects of these parameters were studied for each tool shoulder and tool pin profile. Table 3 shows the tool geometries and process parameters that were used to perform the FSSW joints during this investigation. Figure 4 shows the FSSW process for AA6061-T6 aluminum alloy sheets performed during this investigation.

Four FSSW specimens were performed for each welding condition. According to the process parameters shown in Table 3, three specimens were used to evaluate the average value of the tensile shear failure load (TSFL), and the fourth was used for the micro-hardness and the microstructure examinations.

**Figure 5:**
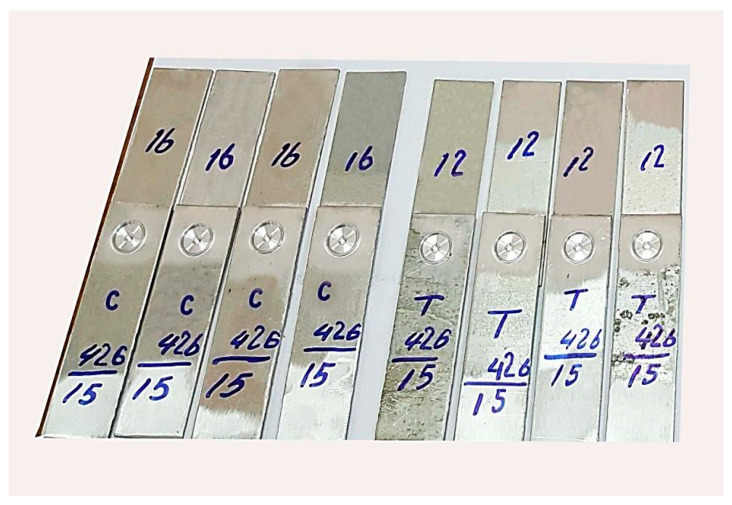
Categorization of performed FSSW specimens according to the welding conditions, tool rotational speed of 426 rpm, 15 s dwell time, shoulder diameters of 12 mm and 16 mm for both the cylindrical and the conical pin tools.

The welded specimens were categorized according to their welding conditions to simplify the welding and testing processes, as shown in Figure 5. Tests were conducted for the performed FSSW lap shear specimens for the mechanical properties and the metallographic examinations. The microstructure examination specimens were cross-sectioned and then wet grinded using different grades of emery SiC paper of 400, 600, 800, 1200, and 2000. Next, the specimens were polished with a diamond compound of 1μm and polishing cloth, and cleaned with water and alcohol. Finally, they were dried with hot air. The specimens were etched with Keller’s reagent solution with composition of (1.5% HCl + 2.5% HNO_3_ + 1% HF + 95% H_2_O) [20], washed with distilled water and alcohol, and dried in an oven.

**Figure 6:**
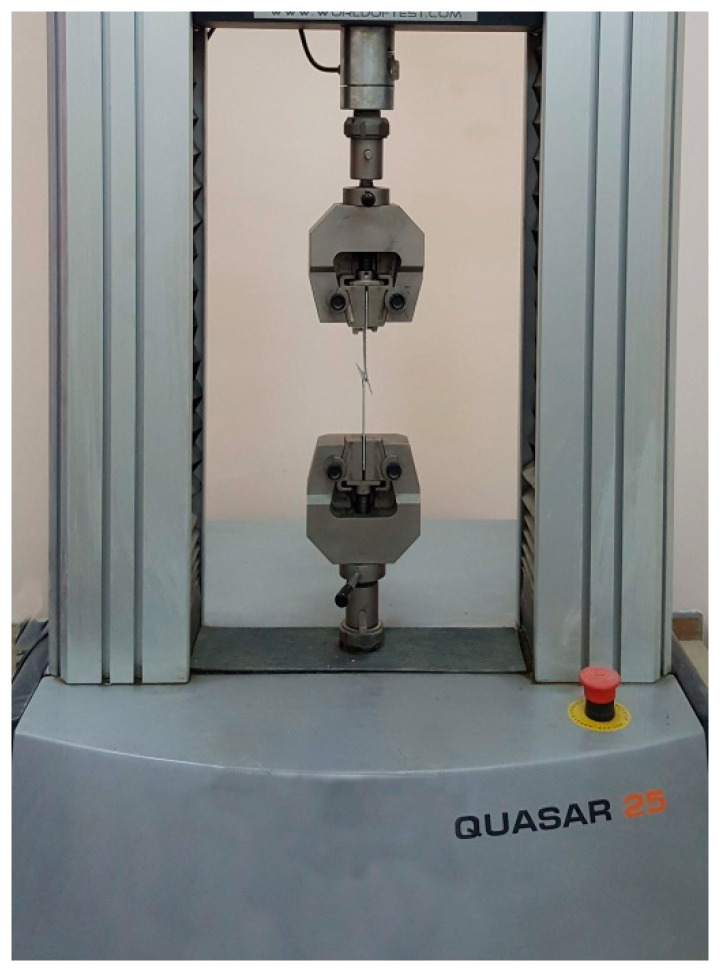
Tensile test of the FSSW lap-shear specimens.

The surface morphology of the FSSW weld region was observed using a computerized optical microscope and the weld features were characterized. The welded specimens were tensile tested to evaluate the TSFL, using QUASAR 25 advancing universal testing device with a cross head speed of 1 mm/min, as shown in Figure 6.

Micro-Vickers hardness test was performed for the cross-section of the FSSW welds that were performed during this study at 0.8 mm below the top of the upper sheet of the FSSW welds, with steps of 0.5 mm, 0.3 kg load, and 15 s dwell time. The influence of the tool geometry on the micro-Vickers hardness was investigated via the microhardness measurements of the cross-section for the upper plates of the FSSW welds, performed in the same process parameters and different tool geometries of 12 mm and 16 mm tool shoulder diameters for both of the SCT and the CPT tools. 

## 3. Results and Discussion

### 3.1. Macro and Microstructure of the Weld Region

**Figure 7:**
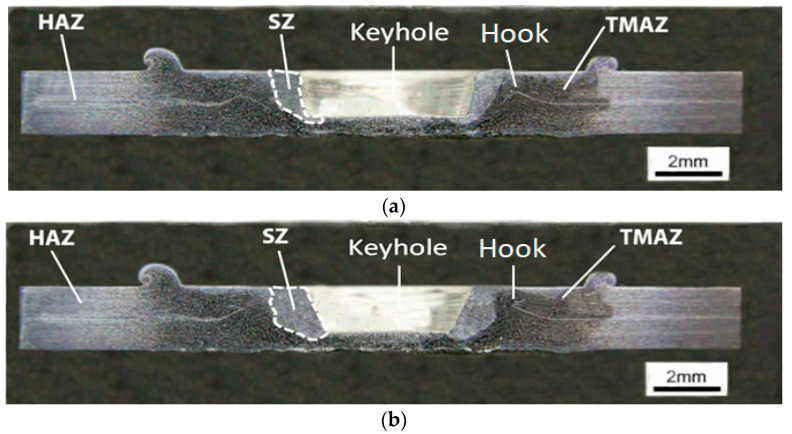

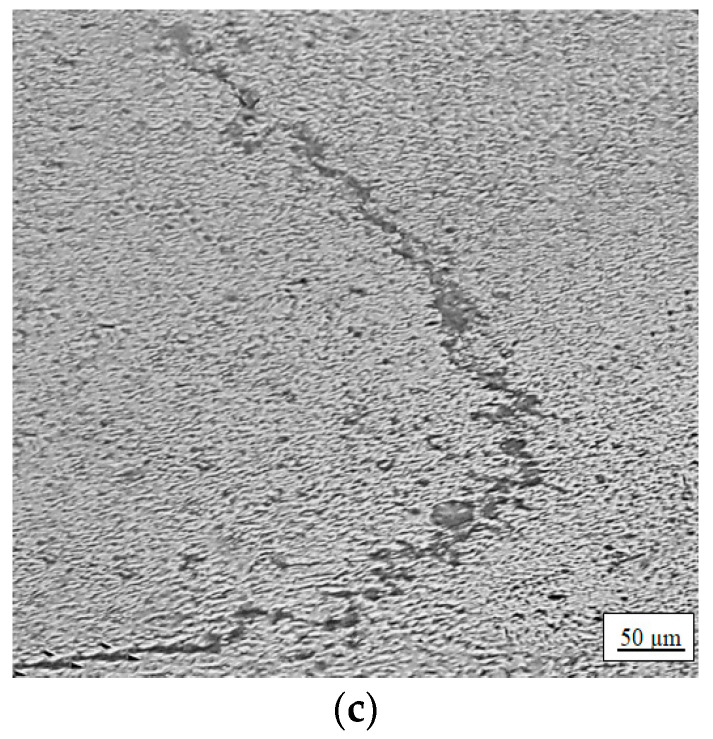
Macrograph of FSSW specimens performed at tool rotational speed of 426 rpm, dwell time of 15 s, and 16 mm shoulder diameter: (**a**) cross-section of a lap-shear specimen performed with the SCT tool; (**b**) cross-section of a lap-shear specimen performed with the CPT tool; (**c**) typical magnified view of the hook observed at specimen (**b**).

Figure 7a,b show macrographs of FSSW specimens produced under the same welding conditions of a tool with a rotational speed of 426 rpm, 15 s dwell time, 16 mm shoulder diameter, but with SCT and CPT tools, respectively. It is clear that the FSSW weld features, such as keyhole, SZ adjacent to the keyhole, and TMAZ, surround the SZ in both specimens. The figures also show the hook defects in both FSSW specimens. 

A keyhole is a common defect in the FSSW technique because it remains at the center of the weld region. Despite the advantages of the FSSW over other traditional welding techniques, the keyhole is aesthetically not preferred. Moreover, corrosion can become more likely within this region. Recently, additional techniques have been developed to reduce the effect of the keyhole, such as the refill-FSSW method and using pinless and consumable welding tools [21]. It can be seen that there is a smaller-sized keyhole in the middle of the specimen and wider SZ formed with the CPT tool compared to the SCT one. Wider SZ is likely to produce higher tensile strength in the FSSW joints. A similar behavior has been reported in previous studies [13,17,22].

As mentioned before, the hook is a common geometrical defect in the FSSW welds in lap configuration. It forms in the interface of the upper and lower sheets of the FSSW lap-shear specimen due to the upward bending resulting from the material flow under the influence of the penetration of the tool during the FSSW process. The hook shape has a significant influence on the weld strength and the failure modes [22,23]. Figure 7a,b show the hook formation from the interface of the upper and the lower sheets of the FSSW lap-shear specimens [22,24]. The hook spread towards the SZ with the use of the SCT tool and towards the periphery of the TMAZ and the SZ when the CPT tool was used. Figure 7c shows a typical magnified view of the hook, observed in the SZ of the cross-section of the FSSW specimen, formed with the CPT tool. It is curved and spreads upwards and to the opposite side of the keyhole. This behavior significantly affects the tensile strength and the failure modes of the FSSW and will be discussed in more detail in next sections of this study.

**Figure 8:**
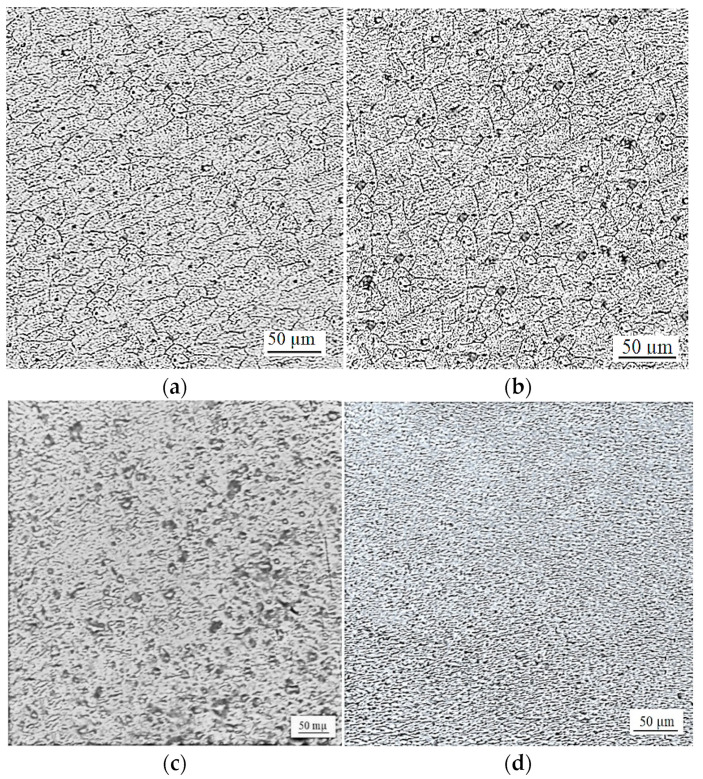
Typical magnified views of FSSW weld specimen formed at 426 rpm, dwell time of 15 s, 16 mm shoulder diameter with cylindrical pin tool: (**a**) BM; (**b**) HAZ; (**c**) TMAZ; (**d**) SZ.

Figure 8a shows the base metal (BM) of the AA6061-T6 aluminum alloy which has equiaxed grains with clear grain boundaries, while Figure 8b–d show typical magnified views of the HAZ, TMAZ, and the SZ, respectively, for the FSSWformed with the CPT tool. The SZ has fine grains resulting from the full dynamic recrystallization produced by means of high frictional heat and high plastic deformation, which are generated using the FSSW tool features, which is why SZ produces such a weld strength. In comparison, TMAZ has lower fine grains fine grains due to the moderate heat input and plastic deformation. Coarser grains observed in HAZ compared to BM are due to HAZ experiencing frictional heat during the FSSW process. There is a clear difference in morphology in the features of FSSW, due to the difference in heat input and material flow.

**Figure 9:**
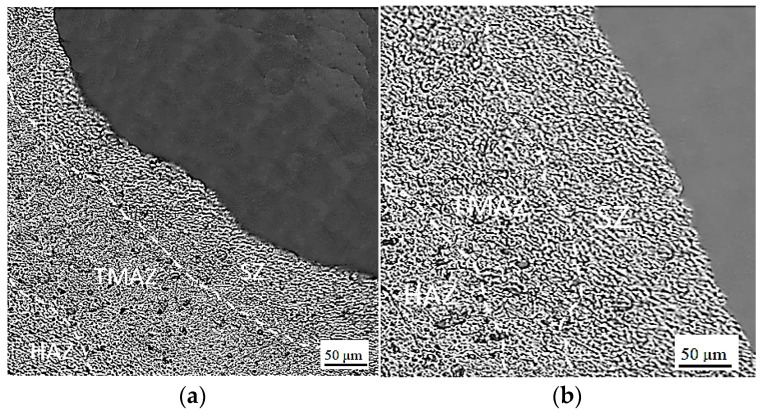
Magnified views of FSSW specimens formed under welding conditions of 426 rpm, 15 s dwell time with different tool geometries: (**a**) 16 mm shoulder diameter and SCT tool; (**b**) 12 mm shoulder diameter and CPT tool.

Process parameters and tool geometry affect frictional heat generation and material flow in the weld nugget during the FSSW process. Consequently, they also influence the morphology of the weld region and control the weld strength. Different process parameters and tool geometries produce different weld morphologies. Figure 9a,b show two FSSW specimensformed at the same welding conditions, but with a 16 mm shoulder diameter with the SCT tool, and a 12 mm tool shoulder diameter with the CPT tool, respectively. As can be seen, there is a difference in grain size and morphology in the FSSW weld features in Figure 9a, compared to the corresponding weld features of the specimen in Figure 9b. Finer grains were observed in the SZ of the specimenformed using the SCT tool compared to the CPT one due to the higher frictional heat generated by the higher shoulder diameter. This confirms the results of Takhakh and Al-khateeb [22] which indicated the significant role of the tool shoulder diameter in controlling the frictional heat generation and dynamic recrystallization, and its effect on the mechanical properties and the microstructure of the FSSW joints. Higher plastic deformation is produced in the SZ by the cylindrical pin tool due to the greater strain hardening and refinement induced by the cylindrical pin than that of the conical pin. This finding agrees with the same concepts in previous studies [13,16,17].

### 3.2. Tensile-Shear Strength

**Figure 10:**
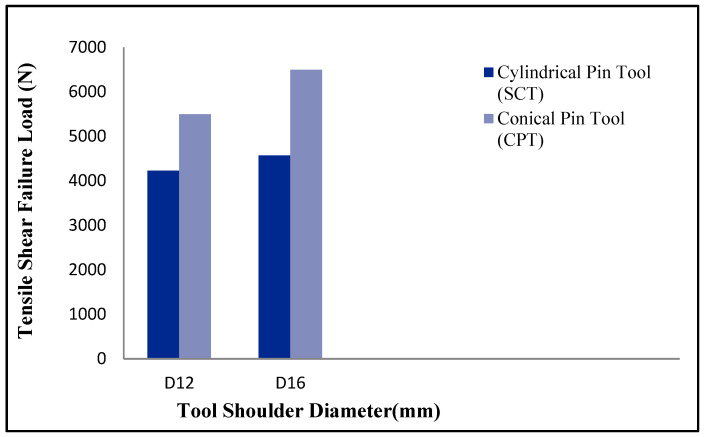
Relationship of TSFL and shoulder diameter for SCT and CPT tools.

The formed FSSW specimens were tensile tested to evaluate the tensile shear strength. Table 4 shows the tensile test results for the corresponding welding conditions, and Figure 10 represents the relationships between the experimental results of the TSFL and the shoulder diameter for both the SCT and the CPT tools.

The test results reported an increase in the TSFL with the increase in the tool shoulder diameter due to the higher generation of frictional heat produced by the 16 mm tool shoulder diameter compared to the 12 mm, with an increase of about 8% in comparison to the SCT tool and an increase of about 18% when the CPT tool was used. Sidhu et al. [14] reported that most of the frictional heat generation during the FSSW process is produced by the tool shoulder compared to the tool pin.

Although finer grains were produced by using the SCT tool, the experimental results reported higher TSFL with the use of the CPT tool than that of the SCT tool—about a 30% increase for the 12 mm tool shoulder diameter and about a 42% increase when the 16 mm shoulder diameter was used. This increase in TSFL can be attributed to the higher bonded area produced during the FSSW process using the CPT tool compared to the SCT tool (Figure 7a,b). The lap-shear failure load of the FSSW joint is directly proportional to the nugget thickness and the weld bonded area [13,17]. Badarinarayan et al. [22], studied this phenomenon and observed a relatively similar behavior when they investigated the effect of other tool pin profiles on the mechanical properties of FSSW welds of 5083 aluminum sheets. Tool pin geometry significantly influences the hook by directly affecting the geometry of its features [22,24,25]. The specimen formed using the SCT tool experienced hook crack propagation through the SZ and towards the keyhole, and failed at a TSFL of 4566 N, while it was 6496 N when the hook was turned upwards and opposite to the keyhole due to the higher material flow and wider ZS achieved by using the tapered pin tool. (Figure 7a,b)

### 3.3. Microhardness

**Figure 11:**
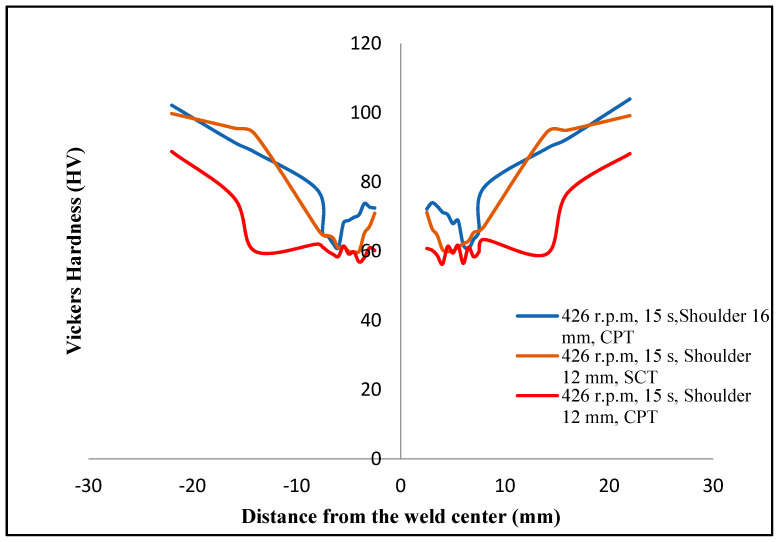
Micro-Vickers hardness distribution profile for FSSW specimens formed using different tool geometries.

Figure 11 represents the Vickers hardness distribution profiles for the FSSW specimens formed with a combination of tool geometries of the SCT and the CPT tool and tool shoulder diameters of 12 mm and 16 mm. 

A typical W-shape of Vickers hardness profiles was observed with a symmetrical profile to the center line of the keyhole for all the examined FSSW specimens. The same general shape has been observed in previous studies [26,27,28]. A gradual decrease in the Vickers hardness was observed in the HAZ due to the coarse grains caused by the frictional heat effect. A sharp hardness decrease was observed in the TMAZ due to the higher heat effect in the periphery of the HAZ and the TMAZ. Then, the hardness increased in the periphery of the TMAZ and the SZ due to the grain refinement produced by the frictional heat and the plastic deformation. A significant increase in the hardness of the SZ compared to the TMAZ was observed due to the finer grains in the SZ obtained by the dynamic recrystallization, as shown in Figure 8c,d.

The specimen examined with the 16 mm shoulder diameter, which indicated a TSFL of 6496 N, appeared to have lower hardness in the SZ compared to the specimen formed under the same welding conditions but with the 12 mm shoulder diameter, which indicated a TSFL of 5496 N. This increase in hardness and an increase in the TSFL are directly related to the shoulder diameter, due to the higher frictional heat generated and the finer grains produced by the 16 mm shoulder diameter compared to the 12 mm one. Figure 11 also shows greater hardness in the SZ observed in specimens formed by the SCT tool, which indicated a TSFL of 4226 N, lower than other specimens performed under the same welding conditions but with the CPT tool, which indicated a higher TSFL of about 5496 N.

Generally, during the FSSW process, the Vickers hardness in the SZ increases due to the strain hardening together with the TSFL, due to the dynamic recrystallization, depending on tool rotational speed [29,30]. In contrast to what was expected, despite the lower TSFL, the greater hardness produced using the SCT tool can be attributed to the higher compaction and higher plastic deformation produced via the SCT tool than that of the CPT tool, due to the higher volume of the cylindrical pin compared to the conical pin when they were compared under similar process conditions. The higher TSFL with lower hardness can be attributed to the greater nugget thickness and bonded area obtained via the conical pin than that of the simple cylindrical pin [13,17]. In a more specific sense, the SCT tool produced greater strain hardening, which led to a higher Vickers hardness compared to the CPT tool. However, the CPT tool produced higher TSFL due to the wider SZ of the FSSW specimen (Figure 7a,b). This finding agrees with those of previous studies [16,29].

### 3.4. Failure Modes

**Figure 12:**
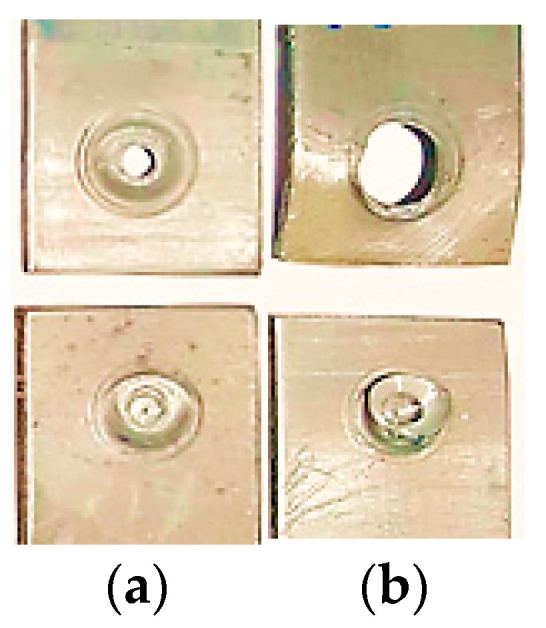
Failure modes of Typical FSSW specimens under tensile shear load formed with different pin tools: (**a**) interfacial failure mode; (**b**) circumferential failure mode.

Figure 12 shows failure modes of FSSW specimens formed under tensile shear load. The specimens were failed always from the weakest points. Table 5 summarizes the failure modes with the corresponding values of the TSFL and the geometry of the tools used to produce the FSSW lap-shear specimens during this study. Two modes of failure were observed under tensile-shear loading, interfacial failure mode and circumferential failure mode. In both modes, the two sheets of the lap-shear specimen were completely separated. In the interfacial failure mode, crack is initiated in the interface of the upper and lower sheets then propagated through the SZ along the original boundary between the upper and lower sheets. The specimen then finally fractured near the periphery of the keyhole and the weld nugget remained in the upper sheet as shown in Figure 12a. This mode of failure was observed at the end of the tensile tests of the FSSW specimens formed with the SCT tool for both 12 mm and 16 mm shoulder diameters at a relatively lower TSFL of 4226 N and 4566 N, respectively.

In the circumferential mode, the crack is initiated in the interface of upper and lower sheets then propagated through the SZ and the TMAZ to the weakest point in the upper sheet towards the shoulder indentation. The specimen is finally fractured at the circumference of the weld nugget in the upper sheet and the weld nugget remained in the lower sheet as shown in Figure 12b. This mode of failure was observed at the end of the tensile tests of the FSSW specimens formed with the CPT tool for both 12 mm and 16 mm shoulder diameters at a relatively higher TSFL of 5496 N and 6496 N, respectively. Worth to be mentioned, the specimens that possessed lower tensile strength failed under the tensile shear load with the interfacial failure mode due to the lower width SZ produced via the SCT tool. On the other hand, the specimens that had higher tensile strength fractured with the circumferential failure mode due to the wider SZ achieved using the CPT tool (Figure 7 and Figure 9). This finding agrees with previous studies [13,17,22].

It seems that the most effective parameter in determining the mode of failure is the shape of the tool pin rather than the shoulder diameter due to the wider SZ associated with the use of the CPT tool in comparison with the SCT one. Previous studies have not investigated the effect of shoulder diameter on the failure modes independently. However, it was investigated while examining the effect of the heat-generating parameters of the FSSW, such as tool rotational speed and dwell time, because the frictional heat has a dominant role in the material flow and determination of the FSSW weld strength, and thus, in specifying the corresponding mode of failure. Many previous studies have investigated the effect of other parameters that are more influential in determining the FSSW failure modes and have reported important conclusions. Paidar et al. [23] reported that the upper sheet thickness near the shoulder indentation is a key factor in determining the mode of failure. They observed that an interfacial failure mode observed at a lower tool rotational speed and a lower plunge depth, while a higher speed produced a circumferential mode regardless of the shoulder plunge depth and the tool geometry. Bilici et al. [16] concluded that a higher shoulder plunge depth decreases upper sheet thickness, and thus decreases the tensile strength and affects the mode of failure. Yuan [6] stated that the tool pin penetration has a significant effect in determining the failure mode of the FSSW joints.

Therefore, the failure mode of the FSSW is a matter of material flow, frictional heat, and upper sheet thickness, depending on the corresponding process parameters, tool rotational speed, dwell time, shoulder diameter, plunge depth. In addition, the tool pin profile affects the failure mode through controlling the nugget thickness and the hook geometry during the FSSW process.

## 4. Conclusions

This study investigated the influence of tool geometry on the mechanical properties and the microstructure in friction stir spot welding (FSSW) of 1.8 mm thickness AA6061-T6 aluminum alloy sheets as a workpiece material. Considered parameters included the tool shoulder diameter and the tool pin profile. The following conclusions were reported:

(1) Finer grains were observed in the SZ of the specimen formed using the 16 mm shoulder diameter and the cylindrical pin tool, compared to the specimen formed using the 12 mm shoulder diameter and conical pin tool. This result is justified by the higher frictional heat generated by the higher shoulder diameter and the higher plastic deformation produced using the cylindrical pin tool.
(2) Higher tensile strength was obtained with the 16 mm shoulder diameter compared with the 12 mm diameter for the SCT and the CPT tools. An increase of about 8% in the case of the SCT and 18% in the case of the CPT were observed.
(3) The experimental results reported higher TSFL with the CPT tool compared to the SCT tool. An increase of about 30% was achieved with the 12 mm tool shoulder diameter and of about 42% when the 16 mm shoulder diameter was used. This increase in TSFL can be attributed to the higher bonded area produced during the FSSW process when using the CPT tool compared to the SCT tool.
(4) Higher hardness in the SZ was reported by the specimen performed with the SCT tool than other specimens performed at the same welding conditions but using the CPT tool. However, the CPT tool produced higher tensile shear strength due to the higher bonded area.
(5) The tool pin profile affects the failure mode through controlling the nugget thickness and the hook geometry.
(6) The FSSW specimens performed with the SCT tool fractured at a relatively lower tensile shear load with the interfacial failure mode. On the other hand, the specimens performed with the CPT tool fractured at a relatively higher tensile shear load with the circumferential failure mode.

## Figures and Tables

**Table 1:** Chemical composition analysis (Wt.%) for AA6061-T6 aluminum alloy.

Element	Si	Fe	Cu	Mn	Mg	Zn	Cr	Ti	Al
(Wt)%	0.80	0.49	0.29	0.12	0.93	0.008	0.26	0.089	Balance

**Table 2:** Mechanical properties of AA6061-T6 aluminum alloy.

0.2% Yield Strength	Tensile Strength (MPa)	Elongation (%)	Hardness (HV)
276	310	12	107

**Table 3:** Tool geometries and process parameters.

Tool Rotational Speed (rpm)	Dwell Time (s)	Plunge Depth (mm)	Plunge Rate (mm)	Shoulder Diameter (mm)	Tool Pin Profile
426	15	0.4	15	12	SCT
426	15	0.4	15	16	SCT
426	15	0.4	15	12	CPT
426	15	0.4	15	16	CPT

**Table 4:** Results of tensile test for FSSW formed specimens.

Shoulder Diameter (mm)	Tool Pin Profile	TSFL (N)
12	SCT	4226
16	SCT	4566
12	CPT	5496
16	CPT	6496

**Table 5:** Failure modes corresponding to tool geometries and tensile shear failure loads.

Shoulder (mm)	Tool Pin	Failure Mode	TSFL (N)
12	SCT	Interfacial	4226
12	CPT	Circumferential	5496
16	SCT	Interfacial	4566
16	CPT	Circumferential	6496

## Data Availability

Data are contained within the article and can be requested from the corresponding author.
